# Breast Cancer DNA Methylation Profiles Are Associated with Tumor Size and Alcohol and Folate Intake

**DOI:** 10.1371/journal.pgen.1001043

**Published:** 2010-07-29

**Authors:** Brock C. Christensen, Karl T. Kelsey, Shichun Zheng, E. Andres Houseman, Carmen J. Marsit, Margaret R. Wrensch, Joseph L. Wiemels, Heather H. Nelson, Margaret R. Karagas, Lawrence H. Kushi, Marilyn L. Kwan, John K. Wiencke

**Affiliations:** 1 Department of Pathology and Laboratory Medicine, Brown University, Providence, Rhode Island, United States of America; 2 Department of Community Health, Center for Environmental Health and Technology, Brown University, Providence, Rhode Island, United States of America; 3 Department of Neurological Surgery, Helen Diller Family Cancer Center, University of California San Francisco, San Francisco, California, United States of America; 4 Department of Biostatistics, Harvard School of Public Health, Boston, Massachusetts, United States of America; 5 Department of Epidemiology and Biostatistics, University of California San Francisco, San Francisco, California, United States of America; 6 Masonic Cancer Center, Division of Epidemiology and Community Health, University of Minnesota, Minneapolis, Minnesota, United States of America; 7 Section of Biostatistics and Epidemiology, Department of Community and Family Medicine, Dartmouth Medical School, Lebanon, New Hampshire, United States of America; 8 Division of Research, Kaiser Permanente, Oakland, California, United States of America; University of Washington, United States of America

## Abstract

Although tumor size and lymph node involvement are the current cornerstones of breast cancer prognosis, they have not been extensively explored in relation to tumor methylation attributes in conjunction with other tumor and patient dietary and hormonal characteristics. Using primary breast tumors from 162 (AJCC stage I–IV) women from the Kaiser Division of Research Pathways Study and the Illumina GoldenGate methylation bead-array platform, we measured 1,413 autosomal CpG loci associated with 773 cancer-related genes and validated select CpG loci with Sequenom EpiTYPER. Tumor grade, size, estrogen and progesterone receptor status, and triple negative status were significantly (*Q*-values <0.05) associated with altered methylation of 209, 74, 183, 69, and 130 loci, respectively. Unsupervised clustering, using a recursively partitioned mixture model (RPMM), of all autosomal CpG loci revealed eight distinct methylation classes. Methylation class membership was significantly associated with patient race (*P*<0.02) and tumor size (*P*<0.001) in univariate tests. Using multinomial logistic regression to adjust for potential confounders, patient age and tumor size, as well as known disease risk factors of alcohol intake and total dietary folate, were all significantly (*P*<0.0001) associated with methylation class membership. Breast cancer prognostic characteristics and risk-related exposures appear to be associated with gene-specific tumor methylation, as well as overall methylation patterns.

## Author Summary

The current standard prognostic indicator for breast cancer is tumor-node-metastasis staging; though, as population-based studies and clinical trials are conducted, molecular characterization of disease is beginning to allow improved markers of prognosis and assist clinicians in choosing the most appropriate therapies. We investigated DNA methylation profiles in over 160 well annotated breast tumor samples and found significant relationships with standard and other known predictors of prognosis, as well as established risk factors for disease: alcohol intake and dietary folate. Recently the United States National Cancer Institute Cancer Biomarkers Research Group articulated a need for a “Strategic Approach to Validating Methylated Genes as Biomarkers for Breast Cancer,” and our work is extremely responsive to this call for a national strategy. Recognizing the increasing use of pre-operative chemotherapy for patients with operable, early-stage disease, there is added complexity in breast cancer staging. Since chemotherapy can considerably decrease tumor size, it is still unclear whether pre-operative or post-operative stage best informs prognosis and treatment decisions for patients electing pre-operative chemotherapy. However, our data clearly illustrate the promise of tumor DNA methylation for augmenting tumor staging and can be attained with minimal tissue in a pre-operative context.

## Introduction

Breast cancer is the most common non-skin cancer among American women. The American Cancer Society's estimates indicate approximately 1.3 million new cases of invasive breast cancer were diagnosed globally in 2007; and nearly 500,000 women died from the disease [1]. Currently, there are over 2.5 million breast cancer survivors in the US, and an estimated $8.1 billion dollars is spent each year on treatment of breast cancer [2].

The principal prognostic indicator currently in clinical use for breast cancer is the tumor-node-metastasis (TNM) stage [3], [4]. Morphological attributes of malignant tumors that influence disease prognosis are the size of the primary tumor (T), presence and extent of regional lymph node involvement (N) and presence of distant metastases (M). Molecular attributes of tumors are also considered in clinical decision-making; loss of hormone receptor expression [5] and increased expression of *ERBB2* [6] have each been associated with poor prognosis. Although numerous recent studies have demonstrated that alterations of DNA methylation in breast cancers are common and may be important etiologic and prognostic markers [7]-[14], large gaps in our knowledge remain. There is a notable lack of studies examining tumor DNA methylation in relation to breast cancer risk factors such as diet or reproductive factors in conjunction with other important tumor markers. Patient exposures such as alcohol and folate intake have potentially strong mechanistic links to epigenetic dysregulation [15]. In addition, recent work in-vitro and in animal models suggest that long term exposure to estrogen may lead to epigenetic effects and altered profiles of DNA methylation [16], [17]. To explore associations of tumor methylation with important tumor and patient characteristics, we analyzed tumors from breast cancer patients in the Kaiser Permanente Division of Research Pathways Study using a large scale methylation array.

## Results

### Unsupervised clustering and locus-by-locus analysis

**Figure 1:**
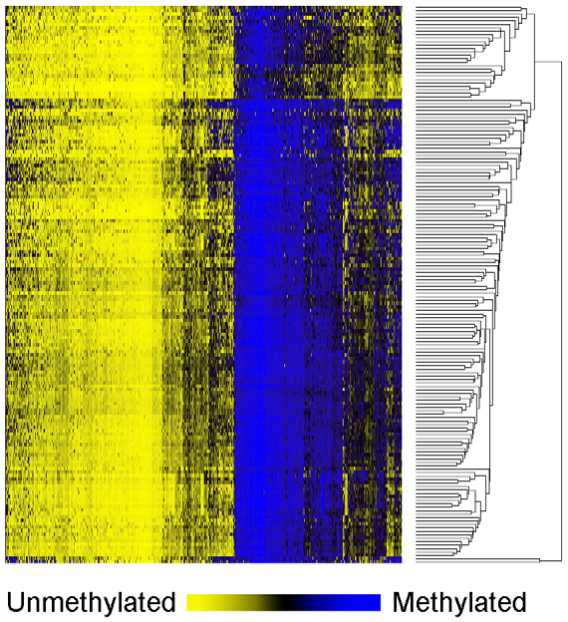
Unsupervised clustering heatmap of CpG methylation in breast carcinomas. Unsupervised hierarchical clustering heat map based on Manhattan distance and average linkage of the 750 autosomal CpG loci with the highest variance. Samples are in rows (n = 162), and CpG loci are in columns. Blue indicates methylated and yellow indicates unmethylated.

Table 1 shows the patient demographic, hormonal, dietary and tumor characteristics for the 162 women overall (and stratified by menopausal status in Table S1). Results of unsupervised hierarchical clustering of the 750 most variable CpG loci indicate the epigenetic heterogeneity of these tumors (Figure 1).

**Table 1:** Patient demographic, hormonal, dietary, and tumor characteristics.

		All cases	
Covariate		n = 162	missing
Age	Range (median)	30–91	1
	Median	59	
	Mean (sd)	59.2 (11.6)	
Race	Caucasian, n (%)	117 (72.7)	1
	African American	13 (8.1)	
	Hispanic	10 (6.2)	
	Asian	10 (6.2)	
	Other	11 (6.8)	
Alcohol (g/day)	Range (median)	0–83.2 (1.8)	8
	Mean (sd)	9.1 (15.4)	
Dietary folate (ug/day)	Range (median)	43.5–1610 (427)	7
	Mean (sd)	459 (213)	
Body mass index	Range (median)	18.5–56.1 (27.4)	2
	Mean (sd)	29.0 (6.7)	
Parity	Nulliparous, n (%)	30 (18.8)	2
	1–2 children	77 (48.1)	
	3–4 children	45 (28.1)	
	5+ children	8 (5.0)	
Histology	Ductal, n (%)	94 (59.1)	3
	Lobular	56 (35.2)	
	Adenocarcinoma	9 (5.7)	
Estrogen receptor	Positive, n (%)	141 (87.6)	1
	Negative	20 (12.4)	
Progesterone receptor	Positive, n (%)	119 (73.4)	1
	Negative	42 (26.6)	
*ERBB2*	Negative, n (%)	134 (85.9)	3
	Positive	22 (14.1)	
Triple negative	No, n (%)	145 (91.2)	3
	Yes	14 (8.8)	
AJCC stage	I, n (%)	95 (59.0)	1
	II	47 (29.2)	
	III	15 (9.3)	
	IV	4 (2.5)	
Tumor Grade	Well differentiated, n (%)	48 (30.0)	
	Moderately differentiated	79 (49.4)	2
	Poorly differentiated	32 (20.0)	
	Undifferentiated	1 (0.6)	
Lymph node status	Positive, n (%)	48 (30.8)	6
	Negative	108 (69.2)	
Tumor Size (mm)	Range (median)	0–135 (14.0)	0
	Mean (sd)	17.4 (15.0)	

In array-wide locus-by-locus analysis the strongest associations of methylation of individual loci (*Q*-values <0.05) were observed for tumor grade (loci n = 209), tumor size (loci n = 74), estrogen receptor status (loci n = 183), progesterone receptor status (loci n = 69), and triple negative status (tumors negative for both estrogen and progesterone receptors as well as *ERBB2*; loci n = 130; Table S2). Together with tumor size, patient lymph node status is used in tumor staging. Among five CpG loci whose methylation was significantly associated (*Q*<0.05) with lymph node status, four (two in *COL1A2*, and one each in *LOX* and *P2RX7*) were also associated with tumor size (*Q*<0.05). Additionally, there was a trend of increased methylation associated with increased tumor size: for all 74 CpG loci that were significantly associated with tumor size (*Q*<0.05) methylation increased with larger tumor size. Similarly, all five CpGs associated with disease-positive lymph nodes had increased methylation in tumors in women with disease-positive lymph nodes. Details of locus-by-locus analyses for tumor grade, size, hormone receptor, and triple negative status (loci with *Q*<0.05) are given in Table S3.

### Array validation

**Figure 2:**
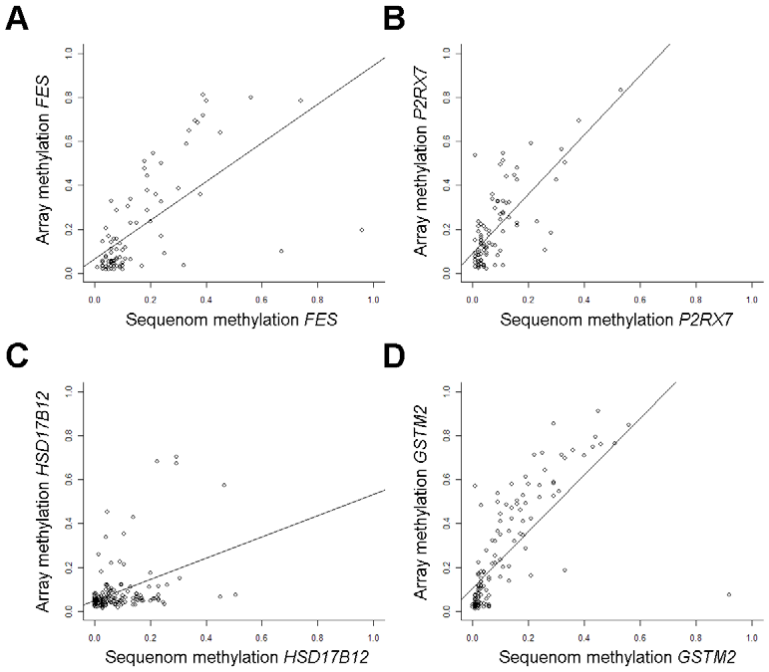
Array methylation is validated by Sequenom EpiTYPER. Results from GoldenGate array methylation values are plotted versus Sequenom EpiTYPER quantitative methylation values. (A) Sequenom *FES* methylation is significantly correlated with GoldenGate methylation average β at the coordinate array CpG (Spearman correlation rho = 0.68, *P* = 1.1E-12, n = 85). (B) Sequenom *P2RX7* methylation is significantly correlated with GoldenGate methylation average β at the coordinate array CpG (rho = 0.65, *P* = 8.6E-12, n = 88). (C) Sequenom *HSD17B12* methylation is significantly correlated with GoldenGate methylation average β at the coordinate array CpG (rho = 0.34, *P* = 5.4E-05, n = 137). (D) Sequenom *GSTM2* methylation is significantly correlated with GoldenGate methylation average β at the coordinate array CpG (rho = 0.83, *P*<2.2E-16, n = 140).

Methylation array validation was performed at CpGs with highly ranked associations from locus-by-locus analysis. The array CpG whose methylation was most significantly increased with increasing tumor stage was in the *FES* gene (Table S3) and array methylation was significantly correlated with Sequenom methylation (rho = 0.68, *P* = 1.1E-12, n = 85; Figure 2A). Promoter CpGs in *P2RX7* and *HSD17B12* had significantly increased methylation (*Q*<0.0001, and *Q* = 0.01 respectively) with increasing tumor size (Table S3) and array methylation at these CpGs were significantly correlated with Sequenom methylation (*P2RX7*; rho = 0.65, *P* = 8.6E-12, n = 88; *HSD17B12*; rho = 0.34, *P* = 5.4E-05, n = 137; Figure 2B and 2C). A promoter CpG in *GSTM2* had significantly increased methylation with increasing tumor grade (Table S3) and array methylation was significantly correlated with Sequenom methylation (rho = 0.83, *P*<2.2E-16, n = 140; Figure 2D). Additionally, in all cases, Sequenom methylation values were significantly associated with respective covariates; tumor stage with *FES* methylation (*P* = 0.05), tumor size with *P2RX7* (*P*<0.005) and *HSD17B12* methylation (*P*<0.02), and tumor grade with *GSTM2* methylation (*P*<0.001). Furthermore, relative mRNA expression of *GSTM2* was significantly decreased among tumors with high array methylation at both CpGs associated with tumor grade (*P*<0.001 and *P*<0.03, Figure S1).

### Clustering of DNA methylation patterns with RPMM

**Figure 3:**
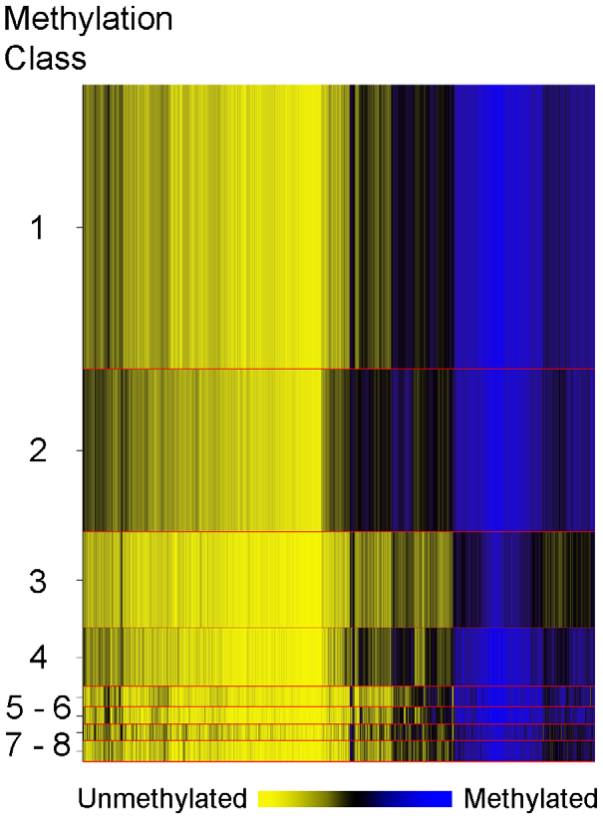
Recursively partitioned mixture model of CpG methylation in breast carcinomas. The figure depicts the results of RPMM. Columns represent CpG sites and rows represent methylation classes. The height of each row is proportional to the number of observations residing in the class, total n = 162. Blue indicates methylated and yellow indicates unmethylated. Methylation classes are numbered one through eight on the left. The color of the columns within each class represents the average methylation of the CpG for that class.

In order to explore overall methylation profiles of these tumors and their potential relationships with patient demographic, tumor and exposure characteristics we applied a modified model-based form of unsupervised clustering known as recursively partitioned mixture modeling (RPMM) [18]. The RPMM resulted in the eight methylation classes (average methylation profiles shown in Figure 3). Patient race was significantly associated with methylation class membership (*P* = 0.015, Table 2), with the majority of African Americans (54%) residing in class 2, and 40% of Hispanic cases residing in class 4. An association between methylation class membership and alcohol consumption approached statistical significance (*P* = 0.07, ever vs. never drinker, Table 2). Both supplemental folic acid intake (µg/day) and total dietary folate (µg/day) had associations with methylation class membership that approached statistical significance (*P* = 0.06 and *P* = 0.08 respectively; Table 2). For both folate variables, cases in methylation class 4 had the lowest intake and cases in methylation class 6 had the highest intake. Of the tumor characteristic variables, only tumor size was significantly associated with overall methylation profile (*P* = 0.0006, Table 2).

**Table 2:** RPMM methylation calss membership by patient demographic and tumor characteristic covariates.

		Class 1	Class 2	Class 3	Class 4	Class 5	Class 6	Class 7	Class 8	Permutation
Covariate		n = 68	n = 39	n = 23	n = 14	n = 5	n = 4	n = 4	n = 5	test *P* *
Age (years)	Mean (sd)	60.8 (11.7)	56.6 (10.4)	61.3 (13.4)	61.4 (10.2)	61.0 (9.0)	57.5 (15.5)	53.8 (10.8)	49.6 (8.0)	0.23
Race	Caucasian, n (%)	53 (79.1)	29 (74.4)	17 (73.9)	7 (50.0)	3 (60.0)	3 (75.0)	2 (50.0)	3 (60.0)	***0.015***
	Hispanic	6 (9.0)	0	2 (7.4)	0	0	1 (25.0)	0	1 (20.0)	
	African American	1 (1.5)	7 (18.0)	2 (7.4)	2 (14.3)	0	0	1 (25.0)	0	
	Asian	3 (4.5)	3 (7.7)	0	4 (28.6)	0	0	0	0	
	Other	4 (6.0)	0	2 (7.4)	1 (7.1)	2 (40.0)	1 (25.0)	1 (25.0)	1 (20.0)	
Alcohol	Never drinker, n (%)	16 (24.6)	13 (36.1)	6 (27.2)	6 (42.9)	0 (0)	3 (75.0)	0 (0)	0 (0)	**0.07**
	Ever drinker	49 (75.4)	23 (63.9)	16 (72.7)	8 (57.1)	5 (100)	1 (25.0)	4 (100)	5 (100)	
Alcohol (g/day)	Mean (sd)	10.2 (16.5)	6.3 (10.0)	11.4 (16.0)	2.3 (4.5)	6.8 (4.0)	8.9 (17.8)	9.1 (11.9)	27.9 (37.9)	0.27
Folic acid (ìg/day)	Mean (sd)	90.2 (68.8)	118 (133)	91.1 (60.2)	55.6 (30.6)	92.9 (37.5)	159 (7.1)	97.7 (52.8)	100 (44.6)	**0.06**
Dietary folate (ìg/day)	Mean (sd)	457 (209)	497 (284)	405 (145)	378 (138)	522 (114)	648 (109)	386 (145)	538 (170)	**0.08**
Stage	Low (1 or 2), n (%)	44 (65.7)	16 (41.0)	12 (52.1)	10 (71.4)	4 (80.0)	2 (50.0)	3 (75.0)	4 (80.0)	0.18
	High (3 or 4)	23 (44.3)	23 (59.0)	11 (47.9)	4 (28.6)	1 (20.0)	2 (50.0)	1 (25.0)	1 (20.0)	
Lymph Node status	Negative	48 (73.8)	22 (57.9)	12 (54.5)	10 (76.9)	5 (100)	4 (100)	3 (75)	4 (80)	0.19
	Positive	17 (26.2)	16 (42.1)	10 (45.5)	2 (23.1)	0	0	1 (25)	1 (20)	
Tumor Size (mm)	Mean (sd)	14.7 (8.9)	24.2 (21.5)	18.3 (12.7)	18.0 (20.0)	13.0 (12.6)	15.3 (13.0)	8.5 (4.9)	9 (9.2)	***0.0006***
Estrogen receptor	Positive, n (%)	60 (89.6)	35 (89.7)	19 (82.6)	11 (78.6)	4 (80.0)	4 (100)	3 (75.0)	5 (100)	0.75
	Negative	7 (10.4)	4 (10.3)	4 (17.4)	3 (21.4)	1 (20.0)	0	1 (25.0)	0	
Parity	Nulliparous, n (%)	13 (19.4)	7 (17.9)	3 (13.6)	2 (14.3)	1 (20.0)	1 (25.0)	1 (25.0)	2 (40.0)	0.95
	1–2 children	29 (43.3)	20 (51.3)	13 (59.1)	5 (35.7)	3 (60.0)	1 (25.0)	3 (75.0)	3 (60.0)	
	3–4 children	22 (32.8)	9 (23.1)	5 (22.7)	6 (42.9)	1 (20.0)	2 (50.0)	0	0	
	≥5 children	3 (4.5)	3 (7.7)	1 (4.5)	1 (7.1)	0	0	0	0	
Oral contraceptive	No, n (%)	21 (31.3)	5 (13.5)	7 (31.8)	6 (42.9)	1 (20.0)	1 (25.0)	0	1 (20.0)	0.35
	Yes	46 (68.7)	32 (86.5)	15 (62.2)	8 (57.1)	4 (80.0)	3 (75.0)	4 (100)	4 (80.0)	

*Running 10,000 permutations.

Tumor grade, histology, and menopausal status were not significantly associated with methylation class.

### Trends of DNA methylation related to alcohol and folate intake

**Figure 4:**
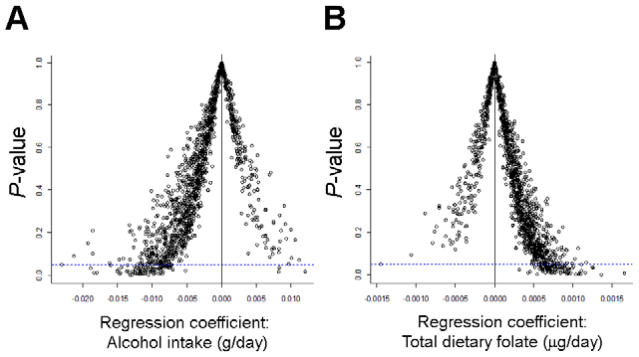
There is an opposite trend for direction of association between breast carcinoma CpG methylation and alcohol intake compared to folate intake. *P*-values for alcohol intake (g/day) and total dietary folate (µg/day) are plotted versus regression coefficients from locus-by-locus analysis of CpG methylation. Horizontal blue dotted line intercepts the y-axis at 0.05 to illustrate significance (before correction for multiple comparisons). The vertical solid black like intercepts the x-axis at zero to illustrate the contrasting trends. (A) There is a trend toward decreased methylation with increasing alcohol intake. (B) There is a trend toward increased methylation with increasing dietary folate.

Associations between alcohol intake and dietary folate and methylation class membership approached statistical significance. While methylation of only one CpG locus (in *IL17RB*) was significantly associated with folate intake in locus-by-locus tests (*Q*<0.05), regression coefficients from univariate locus-by-locus analysis plotted against their respective *P*-values revealed trends in the pattern of methylation for both alcohol and folate intake. Figure 4A illustrates the strong trend for patients with increasing alcohol intake to have negative regression coefficients, indicative of decreased methylation. In contrast, the trend for patients with increasing total dietary folate shows a strong shift to positive regression coefficients, indicative of increased methylation (Figure 4B).

### Multivariate modeling of RPMM classes

**Figure 5:**
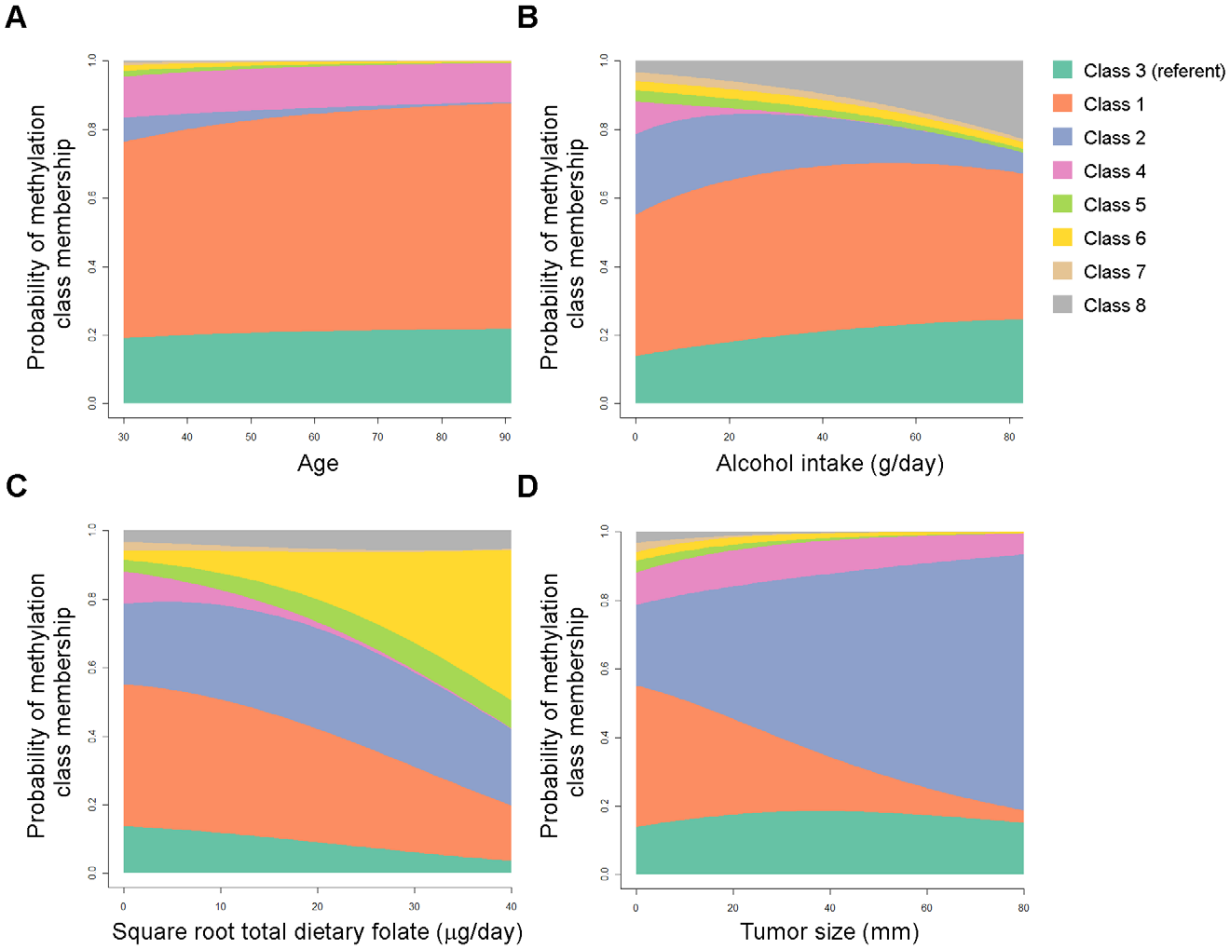
Probability of methylation class membership is significantly associated with tumor size, patient age, alcohol intake, and dietary folate when controlling for potential confounders in a multinomial logistic regression model. Results from a multinomial logistic regression plot the probability of methylation class membership versus covariates controlled for age, race, alcohol consumption, total dietary folate, tumor stage (low vs. high), tumor grade, tumor size, estrogen receptor status, and histology. The referent class (methylation class 3) is on the bottom of the plot in blue-green, remaining classes are plotted in numeric order from bottom to top as shown in the legend. (A) Patient age is significantly associated with methylation class membership (Overall Wald *P*<0.0001), and all methylation classes except class 4 are individually significantly associated with patient age. (B) Alcohol consumption is significantly associated with methylation class membership (Wald *P*<0.0001), and methylation classes 2, 4, 5, and 8 are individually significantly associated with alcohol intake. (C) √Total dietary folate intake is significantly associated with methylation class membership (Wald *P*<0.0001), and all methylation classes are individually significantly associated with total dietary folate. (D) Tumor size is significantly associated with methylation class membership (Wald *P*<0.0001), and all methylation classes except class 4 are individually significantly associated with tumor size.

The relationships between methylation classes and several covariates of interest were then modeled together using multinomial logistic regression in order to adjust for other factors in the model. Patient age, alcohol consumption, total dietary folate, and tumor size were each strongly associated with methylation class membership when controlling for all modeled variables (all Wald *P*-values <0.0001) and complete model details are given in Table S4. Figure 5 displays an illustration of the model results for covariates significantly associated with methylation classes. As alcohol consumption increased, there was an increased probability of cases residing in methylation classes 3 and 8, and a concomitant decrease in the probability of cases residing in classes 2 and 4 (Figure 5B). Increasing total dietary folate intake imparted a striking increase in the probability of membership in class 6, and a decreased probability of class membership in classes 1, 3, 4, and 7 (Figure 5C). The strong association between tumor size and methylation class membership remained after controlling for potential confounders, with the probability of patients being in class 2 increasing from about 20% to about 60% across the span of tumor size from 0 mm to 80+mm (Figure 5D). Accompanying this trend for tumor size were simultaneous decreases in the probability of cases with increasingly large tumors residing in classes 1 and 5–8, while tumor size had less influence on the probability for residing in classes 3 or 4 (Figure 5D).

### Hormone receptor status among postmenopausal cases

Although neither estrogen nor progesterone receptor status were significantly associated with RPMM methylation profiles, large numbers of specific CpG loci had significant methylation associations with these tumor characteristics in locus-by-locus analysis (Table S2 and Table S3). Compared to the overall population of women diagnosed with breast cancer in the Kiaser Permanente Northern California cancer registry from 200–2009, this surgical cohort has a higher prevalence of hormone receptor positivity (78% overall vs. 88% here), particularly among pre-menopausal women's tumors (74% overall vs. 95% here). We therefore stratified on menopausal status, running RPMM on methylation data from post menopausal patients' tumors only (n = 117). This model resulted in eleven methylation classes (Figure S2) and methylation class membership was significantly associated with estrogen receptor status (*P*<0.03), and the association for triple negative tumors approached significance (*P* = 0.07) detailed results available in Table S5.

## Discussion

It is becoming increasingly common to include data on molecular alterations from patient tumor samples into routine clinical practice as a means of improving prognosis and evaluating the predictive power of alterations of interest. As technology improves and population-based studies and clinical trials are conducted, medicine is being ushered into a new era of molecular characterization of disease. Tumor-node-metastasis (TNM) stage is the current prognostic indicator for breast cancer, though several clinical trials are currently under way to investigate the utility of molecular markers [19], and as more patients elect neoadjuvant therapy (specifically pre-operative chemotherapy), improved clinical staging and additional staging tools are poised to have great impact. Most current studies and one commercially available tool (Oncotype DX) are focused on gene expression markers, though the inherent instability of mRNA may make implementation of these strategies challenging outside of major surgical centers or centralized commercial laboratories. In contrast, DNA methylation is a stable mechanism of control of transcription, and the stability of DNA makes it an attractive target for accurate and reproducible assessment. Here we reported that tumor size, a cornerstone of breast cancer prognosis, is associated with tumor DNA methylation profile. In addition, we found that alcohol and folate intake, exposures related to disease risk, are independently associated with tumor DNA methylation profiles. This work sheds light on the relationship between important etiologic exposures and molecular subclasses of disease, extends the evidence for the utility of molecular characterization in tumor staging, and can be accomplished with minimal tissue in a pre-operative context.

The recently updated American Joint Committee on Cancer (AJCC) staging manual for breast cancer does not include additional molecular markers, though the committee acknowledged their consideration of markers such as hormone receptor status and stated that TNM staging “may play increasingly less important roles than understanding the biology of the cancer” [4]. Examining TNM variables we found that overall DNA methylation profile and methylation alterations in dozens of individual CpG loci were significantly associated with tumor size (all increased methylation). In contrast, methylation alterations of only five CpG loci (two in *COL1A2*, and one each in *FAS*, *LOX*, and *P2RX7*) were significantly associated with disease-positive lymph nodes. However, methylation of four of five lymph-node-positive associated CpGs (excepting *FAS*) were also significantly associated with tumor size, suggesting that these phenotypes are mechanistically related, and at least in part manifest via epigenetic alterations. As *FAS* encodes a TNF-receptor involved in regulating apoptosis it is not surprising that methylation-induced silencing of this receptor is associated with disease-positive lymph node status. In addition, hypermethylation of *COL1A2* (collagen type I, alpha 2) has been associated with both proliferation and migration activity in bladder cancer [20], *LOX* is involved in the control of normal collagen deposition [21], and *P2RX7* loss has been linked to morphologic changes in stroma related to altered collagen fibril alignment [22]. Collectively these data suggest that perturbations in collagen and collagen-related genes promote tumor growth and invasion, perhaps by altering the architecture of connective tissues in the tumor microenvironment. In support of this hypothesis, recent work in a mouse model has shown that altered mammary stromal tissue collagen expression significantly increases tumor formation and invasiveness potential [23]. Additionally, Chernov et al. showned that epigenetic alterations in collagen and collagen-related genes allows the deposition of an invasion-promoting collagen matrix in both breast and brain tumor cell lines [24].

The primary objective of TNM staging is to provide a standard prognosis nomenclature for patient care [4], and our results suggest that methylation markers may be a robust proxy for tumor size. Importantly, broader application of neoadjuvant therapy complicates breast cancer staging since chemotherapy can considerably decrease tumor size prior to surgical treatment, and it is still unclear whether clinical or pathologic stage best informs prognosis and treatment decisions [19]. The AJCC has added methodology (yc or ypTNM) for differentiating clinical and pathologic staging; in part, this is from recognition of the increasing use of neoadjuvant therapy for patients with operable, early stage disease [4], [25], [26]. Our data illustrate the promise of tumor DNA methylation for augmenting tumor staging. However, additional study of the relationship between tumor methylation and size in both pretreatment and postoperative samples is necessary. Specifically, the value of methylation to act as an additional marker of size in the neoadjuvant setting should be evaluated in future studies that compare both imaging and pathologically based size determination.

In order to evaluate the predictive power of DNA methylation profiles and individual loci for disease prognosis and recurrence, these patients continue to be followed for these events. Associations between DNA methylation and patient survival have been reported for individual genes such as *GSTP1* and *PITX* [7], [8], [10], though overall DNA methylation profiles, or patterns of methylation at selected CpG loci or genes, may improve predictive power. Well recognized molecular subtypes of breast cancer such as hormone receptor negative and *ERBB2* over-expressing tumors are known to be associated with reduced survival [27], and it will be necessary to extensively examine methylation markers stratified by commonly used molecular tumor markers. However, we did not find significant associations between *ERBB2* status and CpG methylation in our analysis. Nonetheless, other well recognized molecular subtype markers; estrogen receptor, progesterone receptor, and triple negative status were among the covariates with the highest number of significant CpGs from array-wide locus-by-locus analysis. However, hormone receptor status and triple negativity were not associated with methylation profile when modeling all cases. Premenopausal patients' tumors in our surgical cohort had a higher prevalence of hormone receptor positivity compared to the overall population of premenopausal patients diagnosed with breast cancer. In order to address the potential bias this introduced we modeled the methylation profiles of postmenopausal patients' tumors separately and found a significant association between estrogen receptor status and methylation class. Additional study will be needed to better understand the role of hormone receptor and growth factor receptor expression in these tumors as they relate to methylation profile in the context of a patient's menopausal status.

We found significant, independent associations between both alcohol and folate intake and overall tumor DNA methylation profiles when controlling for potential confounders. Folate is a B vitamin that donates its methyl group for homocysteine remethylation to methionine as part of one-carbon metabolism. In turn, methionine is the methyl donor for DNA methylation via S-adenosyl methionine. However, alcohol is known to interfere with folate absorption in the intestine and hepatic release of folate, and hence, supply to tissues [28]. In fact, strong evidence of an etiologic role for alcohol in breast cancer has been reported in multiple meta-analyses of prospective and case-control studies with an excess risk for each alcoholic drink per day of about 10% [29], [30]. In contrast, meta-analysis of prospective studies has not provided clear support for an overall protective association between folate intake and breast cancer risk [31]. Yet, meta-analysis of case control studies of dietary folate, including results from the Shanghai Breast Cancer Study (whose participants are not regular alcohol drinkers) generally support a protective role for folate [31], [32].

While there have been numerous studies of alcohol and folate in relation to risk of breast cancer, investigations of the relationship between these exposures and epigenetic alterations in tumors themselves are scarce. Tao *et al.* reported that the prevalence of breast tumor methylation at *CDKN2A*, *CDH1*, and *RARB* did not differ by folate intake or lifetime alcohol consumption in genotype strata of one-carbon metabolism enzymes methylenetetrahydrofolate reductase (*MTHFR*) and methionine synthase (*MTR*) [33]. Consistent with these findings (and perhaps the lack of similar null results in the literature), we too did not find associations between alcohol or folate and methylation of CpG loci in *CDKN2A*, *CDH1*, and *RARB*. Further, after correcting for multiple comparisons, no CpG loci had significant alcohol-related methylation, and only one CpG locus (in the *IL17RB* promoter) was associated with folate intake. Alone, these results suggested that folate and alcohol intake do not influence tumor DNA methylation. However, plots of regression coefficients indicated strong independent trends for increased folate and reduced alcohol intake associations with increased CpG methylation. Since global, low-level effects of alcohol and folate intake on CpG methylation may not be detectable at individual CpGs in a genome-wide context, we examined the global relationships between alcohol or folate intake and DNA methylation using RPMM methylation classes. Modeling both exposures together revealed highly significant, independent associations between alcohol and folate and DNA methylation profile. Another human cancer for which alcohol is an important etiologic factor is head and neck squamous cell carcinoma, and previous work from our group demonstrated a similar relationship between DNA methylation profiles of these tumors and alcohol consumption [34]. Taken together with the weak mutagenic potential of alcohol [35], these results suggest that a major carcinogenic mechanism of action of alcohol is interference with epigenetic regulation through disruption of one-carbon metabolism.

In summary, we found tumor DNA methylation associated with tumor characteristics predictive of prognosis, and DNA methylation and patient exposures known to be related to disease risk. Additional study is needed to determine the prognostic value of DNA methylation markers. However, the potential clinical utility of tumor-size-related DNA methylation is apparent.

## Materials and Methods

### Study population

The Pathways Study is a prospective cohort study of breast cancer survival actively recruiting women diagnosed with invasive breast cancer from the Kaiser Permanente Northern California (KPNC) patient population since January 2006. Further study details are provided elsewhere [36]. Written informed consent is obtained from all participants before they are enrolled in the study. The study was approved by the IRB of KPNC and all collaborating sites.

### Demographic, hormonal, and dietary factors

During the in-person baseline interview, participants were asked detailed information on family history of cancer and reproductive history, including: age at first full-term pregnancy, number of biological children, breastfeeding, and menopausal status. Additional information was collected on smoking, alcohol use, hormone use (oral contraceptives, hormone replacement therapy), and demographics (age at breast cancer diagnosis, race/ethnicity, household income, education). Self-reported height and weight around diagnosis was obtained to calculate body mass index (BMI, kg/m^2^). Any missing values were supplemented by concurrent information from KPNC electronic medical records.

### Tumor characteristics

Data on estrogen and progesterone receptor status and *ERBB2* expression were obtained from the KPNC Cancer Registry [37]. Tumor size was measured in a uniform manner by participating study pathologists. Data are collected, coded, and added to the KPNC registry approximately four months post-diagnosis to allow for the completion of treatment. For all breast surgical specimens, hormone receptor status and *ERBB2* expression is routinely determined by IHC at the KPNC regional IHC lab, and if the IHC staining for *ERBB2* expression is equivocal (less than 30% strong staining, but more than 10% weak staining), by fluorescence *in situ* hybridization at the KPNC regional cytogenetics lab.

### Study samples

162 tumor specimens from the initial diagnostic biopsy were obtained from the KPNC tumor biorepository for methylation analysis. All tumor specimens were from patients who did not receive neoadjuvant chemotherapy.

### Methylation analysis

FFPE tissue DNA was extracted using the QIAamp DNA mini kit according to the manufacturer's protocol (Qiagen, Valencia, CA). DNA was treated with sodium bisulfite to convert unmethylated cytosines to uracil using the EZ DNA Methylation Kit (Zymo Research, Orange, CA) according to the manufacturer's protocol. Illumina GoldenGate methylation bead arrays were used to simultaneously interrogate 1505 CpG loci associated with 803 cancer-related genes. Bead arrays have a similar sensitivity as quantitative methylation-specific PCR and were run at the UCSF Institute for Human Genetics, Genomics Core Facility according to the manufacturer's protocol and as described by Bibikova et al [38]. GoldenGate array methylation data are publicly available on the Gene Expression Omnibus archive, accession GSE22290.

### Array methylation validation by Sequenom EpiTYPER mass spectroscopy

Array methylation was validated with Sequenom EpiTYPER base-specific cleavage and MALDI-TOF MS of bisulfite treated DNA [39]. EpiTYPER assays were designed for CpG loci both with significant associations between methylation and tumor characteristic variables as well as a high standard deviation of methylation values across samples. One assay (for *COL1A2*) failed the design process. Samples were processed at the UCSF Institute for Human Genetics, Genomics Core Facility. Briefly, PCR with primers located on either side of the CpG sites of interest are transcribed into an RNA transcript and cleaved base specifically. The cleavage products are analyzed by MALDI-TOF MS, and a characteristic mass signal pattern that distinguishes methyl-cytosine from thymine is obtained.

### Gene expression by RT–PCR

Messenger RNA expression was measured using RT-PCR with preamplification using a validated approach [40]. RNA extraction was performed using the RecoverAll (Ambion), with a 16 hour tissue digestion and yields were determined using a Nanodrop spectrophotometer. Samples were concentration-normalized and reverse-transcribed with iScript cDNA synthesis kit (BioRad). Following cDNA synthesis, we performed linear, gene specific preamplification of samples and controls using the TaqMan preamp protocol (Applied Biosystems). Relative expression was measured using a HT7900 real time PCR instrument (Applied Biosystems).

### Statistical analysis

#### Data assembly

Data were assembled with BeadStudio methylation software from Illumina (SanDiego, CA). All array data points are represented by fluorescent signals from both methylated (Cy5) and unmethylated (Cy3) alleles, and methylation level is given by β = (max(Cy5, 0))/(|Cy3|+|Cy5|+100), the average methylation (β) value is derived from the ∼30 replicate methylation measurements. Raw average β values were analyzed without normalization as recommended by Illumina. At each locus for each sample the detection *P*-value was used to determine sample performance; all samples, had detection *P*-values <1e-5 at more than 75% of CpG loci and passed performance criteria. CpG loci with a median detection *P-*value >0.05 (n = 8, 0.5%), were eliminated from analysis. All CpG loci on the X chromosome were excluded from analysis. The final dataset contained 1413 CpG loci associated with 773 genes.

#### Unsupervised clustering

Subsequent analyses were carried out using the R software [41]. For exploratory and visualization purposes, hierarchical clustering was performed using R function hclust with Manhattan metric and average linkage. To discern and describe the relationships between CpG methylation and patient and tumor covariates a modified model-based form of unsupervised clustering known as recursively partitioned mixture modeling (RPMM) was used as described in [18] and as used in [42]. Permutation tests (running 10,000 permutations) were used to test for association with methylation class by generating a distribution of the test statistic for the null distribution for comparison to the observed distribution. For continuous variables, the permutation test was run with the Kruskal-Wallis test statistic. For categorical variables we used the standard chi-square statistic for testing association between two categorical variables.

#### Locus-by-locus analysis

Associations between covariates and methylation at individual CpG loci were tested with a generalized linear model. The β-distribution of average β values was accounted for with a quasi-binomial logit link with an estimated scale parameter constraining the mean between 0 and 1, in a manner similar to that described by Hsuing *et al*. [43]. Array-wide scanning for CpG loci associations with sample type or covariate used false discovery rate estimation and *Q*-values computed by the qvalue package in R [44].

#### Multinomial logistic regression

Multinomial logistic regression was used to model methylation class while controlling for potential confounders. Referent class selection does not affect the underlying interpretation of the model and as class three was neither the largest, nor the smallest class, and was relatively hypomethylated it was chosen as the referent class. Because of the potentially large number of methylation classes, logistic regression coefficients were regularized using a ridge (L2) penalty, with coefficients for a common (non-intercept) covariate across outcome levels shrunk toward zero [34] the tuning parameter was selected by minimizing Bayesian information criterion.

#### Sequenom EpiTYPER methylation and RT–PCR

Spearman correlation coefficients and test *P*-values are reported for correlation between array and Sequenom methylation values. Tests for association between methylation and mRNA expression used relative mRNA expression versus array methylation average β stratified into two groups around 0.5 with the Kruskal-Wallis test statistic.

## Supporting Information

Figure S1 *GSTM2* expression is significantly reduced in tumors with *GSTM2* methylation. Relative mRNA expression values for *GSTM2* are plotted versus array methylation values for two CpGs significantly associated with tumor grade stratified at 0.5. Each box top and bottom edge represents the third and first quartile expression values respectively; box center line represents the median relative expression value. (A) A CpG 153 bases 3′ of the *GSTM2* transcription start site has significantly reduced mRNA expression when methylated (*P*<0.001). (B) A promoter-based CpG 109 bases 5′ of the *GSTM2* transcription start site has significantly reduced mRNA expression when methylated (*P*<0.03).
(0.05 MB DOC)

Figure S2 Recursively partitioned mixture model of CpG methylation in breast tumors from post menopausal patients. The figure depicts the results of RPMM. Columns represent CpG sites and rows represent methylation classes. The height of each row is proportional to the number of observations residing in the class (total n = 117). Blue indicates methylated and yellow indicates unmethylated. The color of the columns within each class represents the average methylation of the CpG for that class.
(0.06 MB DOC)

Table S1 Patient demographic, hormonal, dietary, and tumor characteristics stratified by menopausal status.
(0.04 MB XLS)

Table S2 Summary of results from locus-by-locus CpG methylation versus covariates.
(0.03 MB XLS)

Table S3 Tumor size, grade, hormone receptor, and ERBB2 status are highly associated with CpG methylation in breast carcinoma (n = 162).
(0.12 MB XLS)

Table S4 Multinomial logistic regression of methylation class membership (Class 3 is referent) by patient demographic and tumor characteristics.
(0.04 MB XLS)

Table S5 DNA methylation profiles of post-menopausal patients' breast tumors are significantly associated with estrogen receptor status.
(0.03 MB XLS)

## References

[1] (2007). Global Cancer Facts & Figures 2007..

[2] (2009). Breast Cancer Facts & Figures 2009–2010..

[3] Singletary SE, Connolly JL (2006). Breast cancer staging: working with the sixth edition of the AJCC Cancer Staging Manual.. CA Cancer J Clin.

[4] Edge SB, Byrd DR, Compton CC, Fritz AG, Green FL (2009). AJCC Cancer Staging Atlas: Springer.

[5] McGuire WL (1978). Steroid receptors in human breast cancer.. Cancer Res.

[6] Slamon DJ, Clark GM, Wong SG, Levin WJ, Ullrich A (1987). Human breast cancer: correlation of relapse and survival with amplification of the HER-2/neu oncogene.. Science.

[7] Harbeck N, Nimmrich I, Hartmann A, Ross JS, Cufer T (2008). Multicenter study using paraffin-embedded tumor tissue testing PITX2 DNA methylation as a marker for outcome prediction in tamoxifen-treated, node-negative breast cancer patients.. J Clin Oncol.

[8] Hartmann O, Spyratos F, Harbeck N, Dietrich D, Fassbender A (2009). DNA methylation markers predict outcome in node-positive, estrogen receptor-positive breast cancer with adjuvant anthracycline-based chemotherapy.. Clin Cancer Res.

[9] Novak P, Jensen T, Oshiro MM, Watts GS, Kim CJ (2008). Agglomerative epigenetic aberrations are a common event in human breast cancer.. Cancer Res.

[10] Ronneberg JA, Tost J, Solvang HK, Alnaes GI, Johansen FE (2008). GSTP1 promoter haplotypes affect DNA methylation levels and promoter activity in breast carcinomas.. Cancer Res.

[11] Sinha S, Singh RK, Alam N, Roy A, Roychoudhury S (2008). Frequent alterations of hMLH1 and RBSP3/HYA22 at chromosomal 3p22.3 region in early and late-onset breast carcinoma: clinical and prognostic significance.. Cancer Sci.

[12] Soares J, Pinto AE, Cunha CV, Andre S, Barao I (1999). Global DNA hypomethylation in breast carcinoma: correlation with prognostic factors and tumor progression.. Cancer.

[13] Veeck J, Bektas N, Hartmann A, Kristiansen G, Heindrichs U (2008). Wnt signalling in human breast cancer: expression of the putative Wnt inhibitor Dickkopf-3 (DKK3) is frequently suppressed by promoter hypermethylation in mammary tumours.. Breast Cancer Res.

[14] Veeck J, Noetzel E, Bektas N, Jost E, Hartmann A (2008). Promoter hypermethylation of the SFRP2 gene is a high-frequent alteration and tumor-specific epigenetic marker in human breast cancer.. Mol Cancer.

[15] Mahoney MC, Bevers T, Linos E, Willett WC (2008). Opportunities and strategies for breast cancer prevention through risk reduction.. CA Cancer J Clin.

[16] Bredfeldt TG, Greathouse KL, Safe SH, Hung MC, Bedford MT (2010). Xenoestrogen-Induced Regulation of EZH2 and Histone Methylation via Estrogen Receptor Signaling to PI3K/AKT.. Mol Endocrinol.

[17] Starlard-Davenport A, Tryndyak VP, James SR, Karpf AR, Latendresse JR (2010). Mechanisms of epigenetic silencing of the Rassf1a gene during estrogen-induced breast carcinogenesis in ACI rats.. Carcinogenesis.

[18] Houseman EA, Christensen BC, Marsit CJ, Karagas MR, Wrensch MR (2008). Model-based clustering of DNA methylation array data: a recursive-partitioning algorithm for high-dimensional data arising as a mixture of beta distributions.. BMC Bioinformatics.

[19] Jeruss JS, Mittendorf EA, Tucker SL, Gonzalez-Angulo AM, Buchholz TA (2008). Staging of breast cancer in the neoadjuvant setting.. Cancer Res.

[20] Mori K, Enokida H, Kagara I, Kawakami K, Chiyomaru T (2009). CpG hypermethylation of collagen type I alpha 2 contributes to proliferation and migration activity of human bladder cancer.. Int J Oncol.

[21] Hong HH, Pischon N, Santana RB, Palamakumbura AH, Chase HB (2004). A role for lysyl oxidase regulation in the control of normal collagen deposition in differentiating osteoblast cultures.. J Cell Physiol.

[22] Mayo C, Ren R, Rich C, Stepp MA, Trinkaus-Randall V (2008). Regulation by P2X7: epithelial migration and stromal organization in the cornea.. Invest Ophthalmol Vis Sci.

[23] Provenzano PP, Inman DR, Eliceiri KW, Knittel JG, Yan L (2008). Collagen density promotes mammary tumor initiation and progression.. BMC Med.

[24] Chernov AV, Baranovskaya S, Golubkov VS, Wakeman DR, Snyder EY (2010). Microarray-based transcriptional and epigenetic profiling of matrix metalloproteinases, collagens and related genes in cancer.. J Biol Chem.

[25] Fisher B, Brown A, Mamounas E, Wieand S, Robidoux A (1997). Effect of preoperative chemotherapy on local-regional disease in women with operable breast cancer: findings from National Surgical Adjuvant Breast and Bowel Project B-18.. J Clin Oncol.

[26] Fisher B, Bryant J, Wolmark N, Mamounas E, Brown A (1998). Effect of preoperative chemotherapy on the outcome of women with operable breast cancer.. J Clin Oncol.

[27] Cleator S, Heller W, Coombes RC (2007). Triple-negative breast cancer: therapeutic options.. Lancet Oncol.

[28] Hillman RS, Steinberg SE (1982). The effects of alcohol on folate metabolism.. Annu Rev Med.

[29] Hamajima N, Hirose K, Tajima K, Rohan T, Calle EE (2002). Alcohol, tobacco and breast cancer--collaborative reanalysis of individual data from 53 epidemiological studies, including 58,515 women with breast cancer and 95,067 women without the disease.. Br J Cancer.

[30] Key J, Hodgson S, Omar RZ, Jensen TK, Thompson SG (2006). Meta-analysis of studies of alcohol and breast cancer with consideration of the methodological issues.. Cancer Causes Control.

[31] Larsson SC, Giovannucci E, Wolk A (2007). Folate and risk of breast cancer: a meta-analysis.. J Natl Cancer Inst.

[32] Shrubsole MJ, Jin F, Dai Q, Shu XO, Potter JD (2001). Dietary folate intake and breast cancer risk: results from the Shanghai Breast Cancer Study.. Cancer Res.

[33] Tao MH, Shields PG, Nie J, Marian C, Ambrosone CB (2009). DNA promoter methylation in breast tumors: no association with genetic polymorphisms in MTHFR and MTR.. Cancer Epidemiol Biomarkers Prev.

[34] Marsit CJ, Christensen BC, Houseman EA, Karagas MR, Wrensch MR (2009). Epigenetic profiling reveals etiologically distinct patterns of DNA methylation in head and neck squamous cell carcinoma.. Carcinogenesis.

[35] Dumitrescu RG, Shields PG (2005). The etiology of alcohol-induced breast cancer.. Alcohol.

[36] Kwan ML, Ambrosone CB, Lee MM, Barlow J, Krathwohl SE (2008). The Pathways Study: a prospective study of breast cancer survivorship within Kaiser Permanente Northern California.. Cancer Causes Control.

[37] Oehrli MD, Quesenberry CP, Leyden W (2006). Annual Report on Trends, Incidence, and Outcomes.. Kaiser Permanente Northern California Cancer Registry.

[38] Bibikova M, Lin Z, Zhou L, Chudin E, Garcia EW (2006). High-throughput DNA methylation profiling using universal bead arrays.. Genome Res.

[39] Ehrich M, Nelson MR, Stanssens P, Zabeau M, Liloglou T (2005). Quantitative high-throughput analysis of DNA methylation patterns by base-specific cleavage and mass spectrometry.. Proc Natl Acad Sci U S A.

[40] Li J, Smyth P, Cahill S, Denning K, Flavin R (2008). Improved RNA quality and TaqMan Pre-amplification method (PreAmp) to enhance expression analysis from formalin fixed paraffin embedded (FFPE) materials.. BMC Biotechnol.

[41] R Development CT (2007). R: A Language and Environment for Statistical Computing..

[42] Christensen BC, Houseman EA, Godleski JJ, Marsit CJ, Longacker JL (2009). Epigenetic profiles distinguish pleural mesothelioma from normal pleura and predict lung asbestos burden and clinical outcome.. Cancer Res.

[43] Hsiung DT, Marsit CJ, Houseman EA, Eddy K, Furniss CS (2007). Global DNA methylation level in whole blood as a biomarker in head and neck squamous cell carcinoma.. Cancer Epidemiol Biomarkers Prev.

[44] Storey J, Taylor J, Siegmund D (2004). Strong control, conservative point estimation, and simultaneous conservative consistency of false discovery rates: A unified approach.. J Royal Stat Soc Series B.

