# Supplementary material for: Breast Cancer DNA Methylation Profiles Are Associated with Tumor Size and Alcohol and Folate Intake
Source: PLoS Genet. 2010 Jul 29;6(7):e1001043. doi: 10.1371/journal.pgen.1001043 (PMC2912395; doi:10.1371/journal.pgen.1001043)
Supplement: Figure S1 — GSTM2 expression is significantly reduced in tumors with GSTM2 methylation. Relative mRNA expression values for GSTM2 are plotted versus array methylation values for two CpGs significantly associated with tumor grade stratified at 0.5. Each box top and bottom edge represents the third and first quartile expression values respectively; box center line represents the median relative expression value. (A) A CpG 153 bases 3′ of the GSTM2 transcription start site has significantly reduced mRNA expression when methylated (P<0.001). (B) A promoter-based CpG 109 bases 5′ of the GSTM2 transcription start site has significantly reduced mRNA expression when methylated (P<0.03). (0.05 MB DOC) [file pgen.1001043.s001.doc]

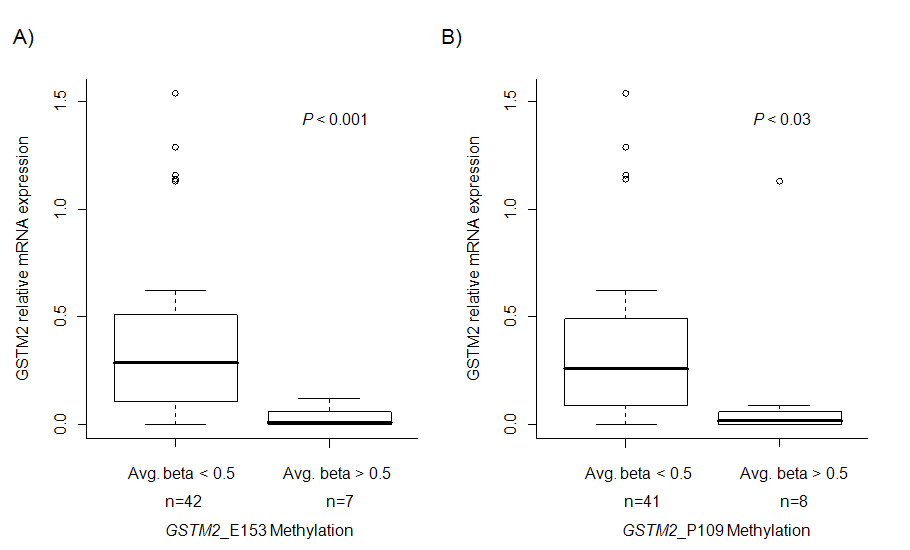


Supplementary Figure 1. *GSTM2* expression is significantly reduced in tumors with *GSTM2* methylation.

Array methylation values stratified at 0.5 are plotted versus relative mRNA expression values for two *GSTM2* CpGs significantly associated with tumor grade. Each box top and bottom edge represents the third and first quartile expression values respectively; box center line represents the median expression value. A) A CpG 153 bases 3’ of the *GSTM2* transcription start site has significantly reduced mRNA expression when methylated (*P* < 0.001). B) A promoter-based CpG 109 bases 5’ of the *GSTM2* transcription start site has significantly reduced mRNA expression when methylated (*P* < 0.03).
