# Supplementary material for: Breast Cancer DNA Methylation Profiles Are Associated with Tumor Size and Alcohol and Folate Intake
Source: PLoS Genet. 2010 Jul 29;6(7):e1001043. doi: 10.1371/journal.pgen.1001043 (PMC2912395; doi:10.1371/journal.pgen.1001043)
Supplement: Figure S2 — Recursively partitioned mixture model of CpG methylation in breast tumors from post menopausal patients. The figure depicts the results of RPMM. Columns represent CpG sites and rows represent methylation classes. The height of each row is proportional to the number of observations residing in the class (total n = 117). Blue indicates methylated and yellow indicates unmethylated. The color of the columns within each class represents the average methylation of the CpG for that class. (0.06 MB DOC) [file pgen.1001043.s002.doc]

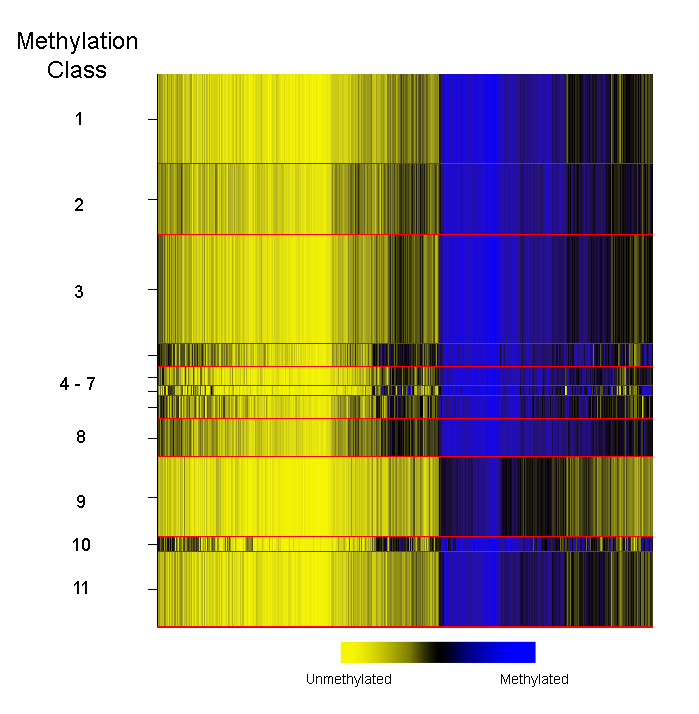


Supplementary Figure 2. Recursively partitioned mixture model of CpG methylation in breast tumors from post menopausal patients.

The figure depicts the results of RPMM. Columns represent CpG sites and rows represent methylation classes. The height of each row is proportional to the number of observations residing in the class (total n=117). Blue indicates methylated and yellow indicates unmethylated. The color of the columns within each class represents the average methylation of the CpG for that class.
